# Treatment decisions in advanced ovarian cancer.

**DOI:** 10.1038/bjc.1989.241

**Published:** 1989-08

**Authors:** M. M. Cody, M. L. Slevin

**Affiliations:** ICRF Department of Medical Oncology, St Bartholomew's Hospital, London, UK.


Br. J. Cancer (1989), 60, 155–156 © The Macmillan Press Ltd., 1989

GUEST EDITORIAL

### Treatment decisions in advanced ovarian cancer

M.M. Cody[1,2] & M.L. Slevin[1]

[1]*ICRF Department of Medical Oncology and* [2]*Department of Psychological Medicine, St Bartholomew's and Homerton Hospitals, London EC1, UK.*

Recent clinical research has greatly contributed to our understanding of the natural history and important prognostic variables associated with ovarian cancer. In comparison, progress in the management of this disease has been disappointingly slow (Slevin, 1986). Ovarian cancer is still the commonest fatal gynaecological cancer in women, accounting for over 3,500 deaths per annum in Great Britain. Although surgical intervention is potentially curative in the early stage, 70–80% of patients present when the disease has spread beyond the ovary due to its initial asymptomatic course (Weiss, 1986). Thus presenting symptoms, even when they are of short duration, usually represent advanced disease.

The current standard approach for stage III ovarian cancer consists of total abdominal hysterectomy, bilateral salpingo-oophorectomy and omentectomy followed by chemotherapy. The actual response rate to chemotherapy and the overall duration of survival, is critically dependent on initial aggressive surgical debulking (Griffiths *et al.*, 1979; Young *et al.*, 1978). Patients with minimal residual disease (less than 2 cm) after surgery are more likely to obtain pathologically complete remissions with induction chemotherapy and this group has the best prospect for long-term survival (Ozols & Young, 1984). Unfortunately, only a minority of women fall into this category. The majority of patients have bulky residual disease after surgery and these women rarely obtain a complete response to chemotherapy. Patients with stage IV disease have a very poor prognosis. In this group the role of debulking surgery is debatable and complete remissions with chemotherapy are rare with the majority of patients relapsing after relatively short periods of remission.

Most patients with stage III or IV ovarian cancer will thus eventually fail primary chemotherapy and second line therapy may be considered. Disease which is refractory to or which relapses after initial chemotherapy is often poorly responsive to conventional chemotherapy. Many of these patients are thus eligible for phase II studies of new drugs. Patients in whom cure is considered highly unlikely and in whom the chance of palliation and prolongation of life is unknown present a difficult problem. A response to chemotherapy may result in relief of symptoms and an improvement in quality of life and may thus be worthwhile even when the treatment is associated with significant side-effects. However, it is likely that only a small minority of patients would achieve such a response with new phase II drugs and it is difficult to predict in advance who these patients will be. The dilemma for the doctor who has to advise the patient in this situation is considerable. He has to decide between prescribing a potentially toxic treatment which may have a negative impact on quality of life with a small chance of remission and recommending no further active treatment other than that aimed at symptom relief. Blackledge *et al.* (1989) have identified predictors of response to second line treatment which should make the doctors task easier. They propose a system of selection criteria for patients entered into phase II studies which might substantially increase the chance of identifying those patients who are likely to respond and suggest that the 'no-hopers' should be excluded from phase II trials of agents with similar activity to first line treatments. Thus patients in this category might not be needlessly exposed to toxic treatments which have little chance of benefit. The authors suggest that instead attention should be focused on symptom relief and ways of improving quality of life. This approach potentially has clear advantages over the current system of entering patients into phase II studies without regard to their likelihood of response. It may explain the wide variation in response rates between different institutions using the same phase II drugs in apparently similar groups of patients. It should also enable more appropriate use of limited research resources.

In practice, advising patients in these difficult situations is extremely complex. It is well known that patient satisfaction is not necessarily dependent on objective tumour response. For some patients, the hope of survival, no matter how slight, the reassurance of more frequent contact with the medical staff, the idea that although treatment may not help themselves but may advance medical science generally, more than compensates for the toxic side-effects or the general inconvenience associated with the therapy. For example, in a study of patients' attitudes to chemotherapy for metastatic gastrointestinal

Correspondence: M. Cody.
Received 9 March 1989.

cancer, 19 out of 25 patients expressed satisfaction with the treatment at 3 months, although only six patients had achieved remission (Gough *et al.*, 1981). Denying patients treatment when they desire it may lead to an increase in psychological morbidity. In a study of depressive illness and lung cancer, depressed patients in the treatment 'observation' group were the most profoundly depressed in the whole group. Many of the patients in this group who were not formally depressed appeared 'puzzled and unhappy'. In part this may have been due to less frequent medical contact and receiving too little information about their illness. Patients in the treatment groups expressed widespread appreciation of active treatment by radiotherapy or chemotherapy even if these treatments had done little to improve physical symptoms or had produced unpleasant side-effects (Hughes, 1985).

Recent research indicates that many patients are willing to undergo intensive treatment even in the knowledge that a favourable outcome is unlikely. In a study asking patients who were about to receive chemotherapy what chance of benefit would make intensive chemotherapy worthwhile, 53% of 106 patients were willing to have intensive treatment for a 1% chance of cure and 42% would accept the same treatment for only 3 months' prolongation of life. There was no significant change in their responses three months after they had received chemotherapy (Slevin *et al.*, 1988). Although one can quite validly question whether patients under stress are capable of making rational, objective decisions, this study nevertheless demonstrates the strength of the will to live in patients with advanced cancer. Reluctance to treat these people may, in some cases, lead to resentment, disappointment and perhaps despair. This information puts the doctor in a difficult position. On the one hand the side-effects and cost of cytotoxic chemotherapy make it extremely inappropriate placebo treatment. On the other hand the doctor does not want to remove hope by giving the patient the feeling that nothing more can be done.

Decisions regarding treatment in recurrent advanced ovarian carcinoma are optimally made collaboratively, after frank and open discussion with the patient in the company of her spouse or partner. Such a discussion must of necessity focus on the seriousness of the situation and the limited chance of further treatment prolonging life. It is essential to ensure that the patient understands what further active treatment might realistically achieve and that the major side-effects and the likely effects on quality of life are fully explained. The patient must then evaluate whether the benefits are worth the costs in the light of her previous experience of chemotherapy, her present quality of life and her value system generally. Patients also need to feel that whatever treatment decision is reached, they will continue to have support and will not be abandoned by their doctors. Explaining to patients that new treatments might become available (however unlikely this may be) is an important component of maintaining hope. The extent to which patients wish to have detailed information about their illness and to participate in decision-making is extremely variable. The injudicious involvement of all patients in the decision-making process is likely to lead to heightened anxiety and doubt among some individuals in an already fraught situation. Although most patients like to be informed about their illness, research indicates that at least one-third prefer to leave treatment decisions to their doctors alone (Cassileth *et al.*, 1980). It is therefore wise to be guided by the patients verbal and non-verbal cues in clinical practice.

There is undoubtedly a need for a more rational approach to the selection of patients to be entered into phase II trials in ovarian cancer. The work of Blackledge *et al.* (1989) represents a welcome attempt to move in this direction. It is important that these predictors of response are verified in other groups of ovarian cancer patients. The question that remains to be answered is what to do for those patients who require further treatment but who are unlikely to respond to phase II drugs. It may be appropriate, as suggested by Blackledge *et al.*, to offer these patients entry into trials of novel agents with completely different activity. When making decisions of this nature, it is important to try to achieve the critical balance between giving sufficient information to the patient to enable them to make individual choices while at the same time being aware of the difficulty many people have in accepting that no further realistic treatment is available.

### References

BLACKLEDGE, G., LAWTON, F., REDMAN, C. & KELLY, K. (1989). Response of patients in phase 2 studies of chemotherapy in ovarian cancer: implications for patient treatment and the design of phase 2 trials. *Br. J. Cancer*, **59**, 650.

COATES, A., ABRAHAM, S., KAYE, S.B. and 4 others (1983). On the receiving end: patient perception of the side-effects of cancer chemotherapy. *Eur. J. Cancer Clin. Oncol.*, **19**, 203.

GOUGH, I.R., FURNIVAL, C.M. & BURNETT, W. (1981). Patient attitudes to chemotherapy for advanced gastro-intestinal cancer. *Clin. Oncol.*, **7**, 5.

GRIFFITHS, C.T., PARKER, L.M. & FULLER, A.F. (1979). Role of cytoreductive surgical treatment in the management of advanced ovarian cancer. *Cancer Treat. Rep.*, **63**, 235.

HUGHES, J.E. (1985). Depressive illness and lung cancer. II. Follow-up of inoperable patients. *Eur. J. Surg. Oncol.*, **11**, 21.

OZOLS, R.F. & YOUNG, R.C. (1984). Chemotherapy of ovarian cancer. *Semin. Oncol.*, **11**, 251.

SLEVIN, M.L. (1986). Ovarian cancer. In *Randomized Trials in Cancer: a Critical Review by Sites*, Slevin, M.L. & Staquet, M.J. (eds). Raven Press: New York.

SLEVIN, M.L., PLANT, H., STUBBS, L. and 2 others (1988). Balancing the possible benefits against the risk of cytotoxic chemotherapy – patients' and doctors' decisions. British Association of Cancer Research abstract. *Br. J. Cancer*, **58**, 266.

WEISS, G.R. (1986). Second-line chemotherapy for ovarian cancer. *Clin. Obstet. Gynecol.*, **29**, 665.

YOUNG, R.C., CHABNER, B.A., HUBBARD, S.P. *et al.* (1978). Advanced ovarian adenocarcinoma: a prospective clinical trial of melphalan (L-PAM) versus combination chemotherapy. *N. Engl. J. Med.*, **299**, 1261.


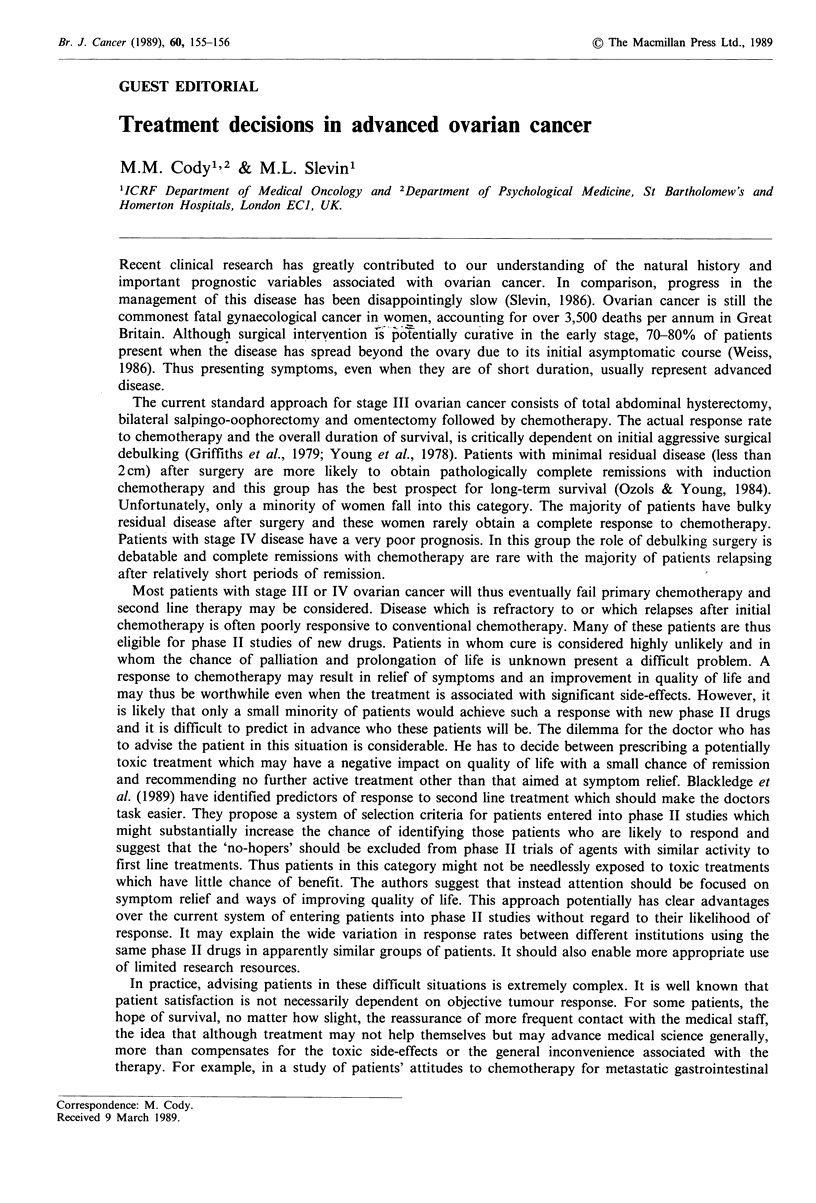

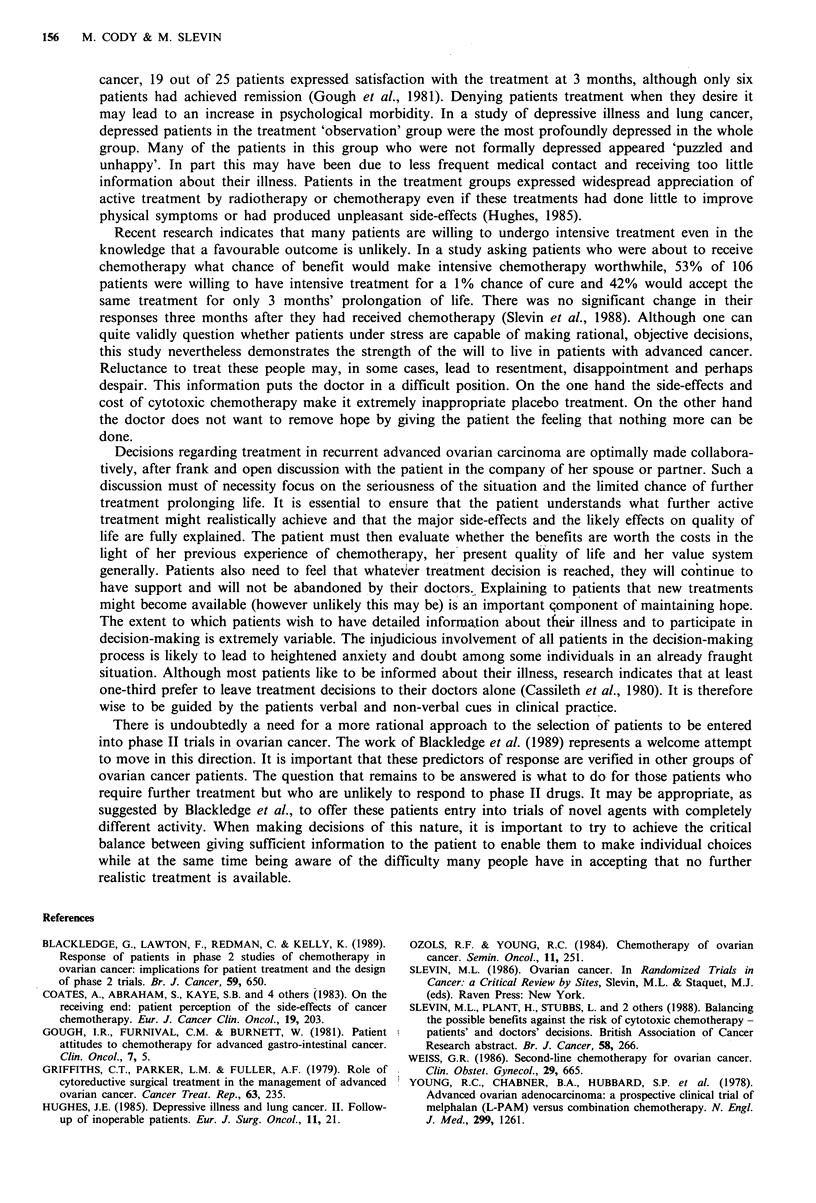


## References

[OCR_00127] Blackledge G., Lawton F., Redman C., Kelly K. (1989). Response of patients in phase II studies of chemotherapy in ovarian cancer: implications for patient treatment and the design of phase II trials.. Br J Cancer.

[OCR_00133] Coates A., Abraham S., Kaye S. B., Sowerbutts T., Frewin C., Fox R. M., Tattersall M. H. (1983). On the receiving end--patient perception of the side-effects of cancer chemotherapy.. Eur J Cancer Clin Oncol.

[OCR_00138] Gough I. R., Furnival C. M., Burnett W. (1981). Patient attitudes to chemotherapy for advanced gastro-intestinal cancer.. Clin Oncol.

[OCR_00143] Griffiths C. T., Parker L. M., Fuller A. F. (1979). Role of cytoreductive surgical treatment in the management of advanced ovarian cancer.. Cancer Treat Rep.

[OCR_00148] Hughes J. E. (1985). Depressive illness and lung cancer. II. Follow-up of inoperable patients.. Eur J Surg Oncol.

[OCR_00152] Ozols R. F., Young R. C. (1984). Chemotherapy of ovarian cancer.. Semin Oncol.

[OCR_00167] Weiss G. R. (1986). Second-line chemotherapy for ovarian cancer.. Clin Obstet Gynecol.

[OCR_00171] Young R. C., Chabner B. A., Hubbard S. P., Fisher R. I., Bender R. A., Anderson T., Simon R. M., Canellos G. P., DeVita V. T. (1978). Advanced ovarian adenocarcinoma. A prospective clinical trial of melphalan (L-PAM) versus combination chemotherapy.. N Engl J Med.

